# Human Health Risk and Bioaccessibility of Toxic Metals in Topsoils from Gbani Mining Community in Ghana

**DOI:** 10.5696/2156-9614-9.22.190602

**Published:** 2019-05-20

**Authors:** Godfred Darko, Kwadwo Owusu Boakye, Marian Asantewaa Nkansah, Opoku Gyamfi, Eugene Ansah, Lily Lisa Yevugah, Akwasi Acheampong, Matt Dodd

**Affiliations:** 1 Department of Chemistry, Kwame Nkrumah University of Science and Technology, Kumasi, Ghana; 2 Department of Geomatic Engineering, Kwame Nkrumah University of Science and Technology, Kumasi, Ghana; 3 School of Environment and Sustainability, Royal Roads University, Victoria, Canada

**Keywords:** soil contamination, environmental pollution, small-scale artisanal mining, bioaccessibility

## Abstract

**Background.:**

Anthropogenic activities such as artisanal mining pose a major environmental health concern due to the potential for discharge of toxic metals into the environment.

**Objectives.:**

To determine the distribution and pollution patterns of arsenic (As), iron (Fe), nickel (Ni), cobalt (Co), chromium (Cr), manganese (Mn), copper (Cu) and zinc (Zn) in the topsoil of a mining community in Ghana, along with potential human health risks and *in vitro* bioaccessibility.

**Methods.:**

Concentrations of metals were determined using X-ray fluorescence techniques and validated using inductively coupled plasma-mass spectrometry.

**Results.:**

Concentrations of the metals in topsoil were in the order of magnitude of Cu (31.38 mg/kg) < Ni (45.39 mg/kg) < As (59.66 mg/kg) < Cr (92.87 mg/kg) < Zn (106.98 mg/kg) < Mn (1195.49 mg/kg) < Fe (30061.02 mg/kg). Geo-statistical and multivariate analyses based on hazard indices including contamination, ecological risks, geo-accumulation, and pollution load suggest that the topsoils are contaminated in the study area. The potential ecological risk index (PERI) showed high ecological risk effects (PERI=269.09), whereas the hazard index (1×10^−7^) and carcinogenic risk index (1×10^−5^) indicated low human health risks. Elevated levels of As, Cr, Ni, and Zn were found to emanate from anthropogenic origins, whereas Fe, Mn, and Cu levels were attributed mainly to geological and atmospheric depositions. Physicochemical parameters (pH, electrical conductivity and total organic carbon) showed weak positive correlations to the metal concentrations. Elemental bioaccessibility was variable, decreasing in the order of Mn (35± 2.9%) > Cu (29± 2.6%) > Ni (22± 1.3%) > As (9± 0.5%) > Cr (4± 0.6%) > Fe (2± 0.4%).

**Conclusions.:**

Incorporation of in-vitro bioaccessibility into the risk characterization models resulted in a hazard index of less than 1, implying low human health risks. However, due to accumulation effects of the metals, regular monitoring is required.

**Competing Interests.:**

The authors declare no competing financial interests.

## Introduction

Metals found in the environment generally originate from natural processes such as weathering of rocks, atmospheric deposition, or from anthropogenic sources, but their distributions are influenced by the properties of the metals and physicochemical factors of soil such as organic matter content and pH. The widespread distribution of metals in the environment is explained by the stability of the forms in which they occur. Whereas low concentrations of essential metals such as copper (Cu), iron (Fe), manganese (Mn), and zinc (Zn) are beneficial for the growth and maintenance of the human body, toxic metals (cadmium (Cd), mercury (Hg) and lead (Pb)) can have harmful effects on animal and plant life, as well as the environment.^1–3^ Some toxic metals such as Cd, Pb, and Hg are widely used in the manufacturing, mining, agricultural and medical fields and are eventually discharged into the environment.^4–6^

Accumulation of excess amounts of metal contaminants in the environment threatens the health of plants and animals because these metals exert biological effects on all life forms.^7^,^8^ Metal pollution in soils is of concern to researchers and regulatory agencies because most metals have adverse health effects.^9^ Long term exposure to metals can result in reduced intelligence in humans, DNA damage, and memory impairment.^10^,^11^ The toxic effects of metals are normally defined by their nature. For example, mercury and lead affect almost every human organ, arsenic is known to be a human carcinogen, and cadmium affects the kidneys and lungs.^12^

Anthropogenic activities, including artisanal mining, pose a major environmental health risk due to the potential for discharge of toxic metals into the environment. There has been an upsurge of artisanal mining activities which have polluted almost all major rivers in the country as well as topsoil in the affected communities.^13^

Bioavailability depicts the amount of a contaminant absorbed into systemic circulation through major exposure pathways (dermal, oral and inhalation).^14^ Soil characteristics such as conductivity, pH, texture and organic matter content may influence metal bioavailability.^15^ Metal speciation also affects bioavailability. Oral bioavailability is best assessed through in vivo studies, however, due to the cost, time and ethical concerns associated with in vivo testing of animals, *in vitro* digestion models using physiologically-based extraction tests which mimic the activities in the human digestive track have been developed.^16^ Incorporation of physiologically-based extraction tests in risk calculations improves the accuracy of the assessment.^17^,^18^

This study was undertaken to assess the potential sources and distribution of toxic metals in the community of Gbani, an area in Ghana where mining is predominantly practiced, estimate the extent of contamination using hazard indices, and determine the potential health risk associated with incidental ingestion of metal-contaminated soils using *in vitro* bioaccessibility assay as a substitute for oral bioavailability.

## Methods

**Figure 1:**
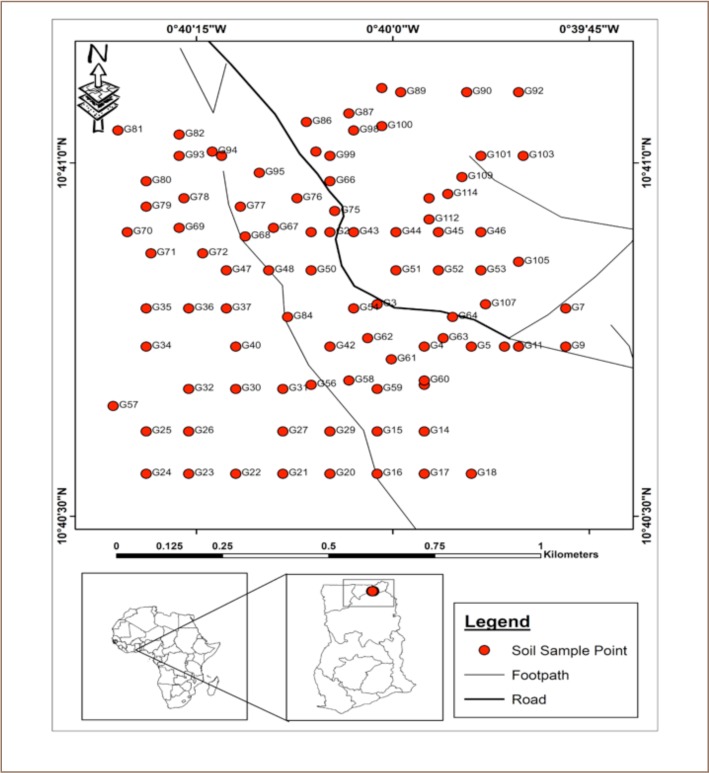
Map of Gbani showing the sampling locations

Gbani (Global Positioning System (GPS) coordinate: 11.7351035, -2.392887) is in the Talensi District of the Upper East Region of Ghana (*Figure 1*). The community is noted for mining activities, where artisanal small-scale gold mining activities have grown rapidly. The district is one of the poorest areas in Ghana and artisanal small-scale gold mining is estimated to directly employ over 10,000 people there.^19^,^20^ Vegetation in the district is mainly savannah woodland interspersed with short, droughtresistant trees and grasses. Gbani has a population of approximately 3000 people living in about 400 houses in a community where artisanal gold mining activities, the mainstay of the community, are widely scattered.

**Abbreviations**
*CF*: Contamination factor
*EF*: Enrichment factor
*ICP-MS*: Inductively coupled plasmamass spectrometer
*PC*: Principal component
*PERI*: Potential ecological risk index
*PLI*: Pollution load index
*XRF*: X-ray fluorescence

### Soil sampling

Soil sampling was conducted from June–October 2016. The entire community was mapped on a 100 × 100 m grid net and samples taken at the points of intersections of the coordinates (*Figure 1*). At the points where sampling was not possible due to interference such as tarred roads, rivers and houses, the sampling points were adjusted by moving 2 m away to either the left or right of the actual sampling point, but within the same sampling grid cell.

About 500 g of soil samples were taken from the top 0–10 cm depths using plastic trowels. The topsoil from 0–10 cm is the layer human beings are most exposed to during their daily activities and provides better human exposure risk estimates.^21^,^22^ A total of 94 soil samples were taken across the community. Duplicate samples were taken after every 5 sampling points. All glassware and apparatus for taking samples were initially soaked overnight in dilute HNO_3_, then cleaned with detergent and deionized water and dried. Cross contamination of the digging equipment was minimized by scrubbing the digging devices with deionized water and detergents after each sample was collected. Soil samples were placed into sealed and well-labeled Ziploc bags and transported to the laboratory for analysis.

### Total metal concentration analysis

Soil samples were air-dried in the sun to ensure that they were free from moisture. Each of the dried soil samples was sieved to < 250 μm using a USA Standard Testing Sieve ASTM E11. Two hundred and fifty (250) μm is considered to be most representative of the particle size that adheres to children's hands and therefore most likely to be ingested through hand-to-mouth (pica) behavior.^23^ Soil samples were screened using a Niton XL3 GOLDD+ X-ray fluorescence (XRF) analyzer. The soil analysis followed the United States Environmental Protection Agency (USEPA) Method 6200; field portable X-Ray fluorescence spectrometry for the determination of elemental concentrations in sediment and soil protocol.^24^ The XRF was calibrated using NIST 2711 standard and system checked each day prior to use.^25^ An aliquot of the sieved sample (~2 g) was placed in a small (approximately 30 mm) polyethylene container so it was three quarters full and sealed at both ends using a mylar film. It was then placed in the XRF shroud and scanned for 180 seconds.

Following XRF analysis, 20 samples representing the different settings in the area were analyzed using an Agilent 7800 inductively coupled plasma-mass spectrometer (ICP-MS) as confirmatory samples for the XRF analysis. The sieved soil samples were analyzed for total metals by ICP-MS after modified aqua regia digestion. Briefly, 1 g sample was added to 10 mL of 1:1:1 hydrogen chloride-nitric acid-water mixture. The mixture was digested at 95°C for 1 hour in a heating block. The sample was made to volume with dilute hydrogen chloride and analyzed using the ICP-MS based on USEPA SW 846 test method 6020B.^26^

### Total organic carbon

Soil samples were analyzed for total organic carbon using the loss on ignition method.^27^,^28^ An aliquot of 1 g the soil sample was weighed directly into a crucible and placed in a preheated oven at 105°C for two hours. The samples were removed and cooled to room temperature in a desiccator before weighing. The samples were then placed in Thermolyne muffle furnace, pre-heated to 550°C for 4 hours. The samples were removed from the furnace and cooled to room temperature (overnight) in a desiccator and re-weighed. Replicate measurements were carried out to ensure reproducibility of results. Organic matter content was calculated as the difference between the initial and final weights divided by the initial sample weight multiplied by 100%.

### Soil pH and electrical conductivity

Soil pH and electrical conductivity were determined using a multiparameter probe (Oakton waterproof multiparameter PCSTestr 35). The pH and electrical conductivity measurements were performed by mixing 20 g of the dry soil in 40 mL of distilled water (1:2 ratio of dry soil to distilled water). The suspension of water and soil particles was allowed to settle for 1 hour, following which the pH and electrical conductivity values of the supernatant liquid were read on the instrument.

### *In vitro* bioaccessibility assay

The extraction protocol was based on the standard operating procedure for an *in vitro* bioaccessibility assay for lead and arsenic in soil.^29^,^30^ In brief, the sieved soil sample was weighed by difference (1.00±0.05 g) into a 125 mL acid cleaned high density polyethylene bottle. An aliquot of the extraction fluid (100±0.5 mL) consisting of 30 g/L glycine adjusted to pH 1.5 with concentrated hydrogen chloride was measured and added to the bottle. The pH of the soil/extraction fluid mixture was measured. The bottles were then sealed and placed into an extractor in batches of 8 and rotated end-over-end in a 37±2°C water bath for 1 hour. After the extraction, each extract was drawn directly into a disposable 20 mL plastic syringe with a luer slip. A 0.45 μm cellulose acetate filter was attached to the syringe and the extract was filtered into a clean 20 mL polyethylene scintillation vial. Quality assurance/quality control included a procedure blank, a laboratory control sample and duplicate analysis. The filtered extracts were analyzed for selected metals using an Agilent series 7800 ICP-MS.^26^ Bioaccessibility was calculated by dividing the concentration of metal in the extract by the total metal concentration and expressed as a percentage.

### Statistical analysis

Statistical analysis of the data was performed using Minitab version 20.0 and ProUCL version 5.1. Results from the analyses were tested for distribution using the goodness-of-fit test.^31^ The majority of the data did not follow a normal distribution, and as such nonparametric statistics such as the Wilcoxon Mann-Whitney test were used for quantitative comparisons of metal concentrations and bioaccessibility at the 5% significance level. To allow for a comparison of onsite contamination due to background concentrations, the mean, median, standard deviation and 95 percentile concentrations were also determined. Pearson correlation analyses were used to determine the relationship between physicochemical parameters and metal concentrations.

### Estimation of level of contamination

The extent of metal contamination can be evaluated by either comparing site specific data to background reference data, or through the use of pollution indices and enrichment factors.^32^ In this work, contamination was measured by the use of enrichment and contamination factors, pollution load, geoaccumulation, and potential ecological risk indices.

### Enrichment factor

Enrichment factor (EF) was used to assess the degree of toxic metal pollution from anthropogenic sources and to differentiate between anthropogenic and naturally occurring sources.^33^ Concentration of Fe was used as the normalization reference.^34^ The EF was calculated using Equation 1.

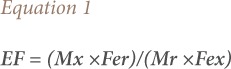
Where Mx and Fex are specified metal and Fe concentrations, respectively, determined in the sample, while Mr and Fer are the concentrations of the specified metal and Fe in a reference material. An EF ≤ 1 means the metal concentration in the soil sample is enriched relative to the average continental crust and surface soils and the source of the metal in the topsoil is likely to be anthropogenic. An EF ≤ 1 indicates that the metal concentration is not enriched and may be from a natural source. An EF=1 indicates that the metal concentration and its reference value are the same.^33^

### Contamination factor

Contamination factor (CF), which is the ratio of the concentration of a metal in the soil to background levels, was used to assess the extent of metal contamination.^35^ The CF was calculated using Equation 2.

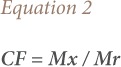
Where Mx and Mr are the concentrations of a metal contaminant in the soil sample and background reference material, respectively. The level of contamination by the metal pollutants in the study area was also assessed using modified degree of contamination.^22^

### Pollution load index

To estimate the overall pollution status of the samples, the pollution load index (PLI) of the metal contaminants was calculated using Equation 3.^5^,^36^

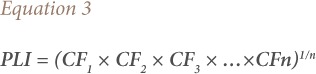
Where CF represents the contamination factor of a metal and n represents a specific metal's contamination factor. The PLI values are used to classify soils as unpolluted (PLI ≤ 1), moderately polluted (PLI = 1–3), highly polluted (PLI = 3–5), or very highly polluted (PLI ≤ 5).^5^

### Potential ecological risk

The potential ecological risk index (PERI) was used to assess the degree of metal pollution in soils based on the CF of metals and the response of the environment to the contaminant (Trf). The PERI was calculated as the sum of individual risk indices using Equation 4.^35^

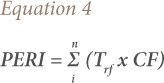



Toxic response factor for metals are in the order of Zn = 1, chromium (Cr) = 2, cobalt (Co) = Cu = Pb = 5, nickel (Ni) = 6, arsenic (As) = 10, Cd = 30 and Hg = 40.^5^,^15^ The degree of ecological risk was classified as PERI ≤ 40 (low risk), 40 ≤ PERI ≤ 80 (moderate risk), 80 ≤ PERI ≤ 160 (considerable risk), 160 ≤ PERI ≤ 320 (high risk), and PERI ≥ 320 (very high risk).^16^,^33^

### Potential human health risk

For incidental soil ingestion, Equation 5 was used to quantify average daily metal intake.^37^

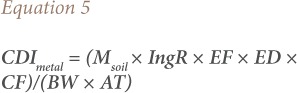
where CDImetal = metal daily intake (mg kg^−1^ day^−1^); Msoil = metal concentration in soil (mg kg^−1^); IngR = ingestion rate of soil (mg day^−1^); EF = exposure frequency (day year^−1^); ED = exposure duration (year); BW = body weight (kg); AT = averaging time (days); and CF = conversion factor (10^−6^ kg mg^−1^). The above equation assumes 100% bioavailability of the ingested metal. However, metal bioavailability is dependent on metal speciation and soil properties such as pH, texture, and organic matter. Equation 5 can therefore be adjusted to include a relative bioavailability factor as follows:^16^

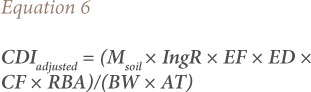
Where RBA = relative bioavailability (unitless).

Carcinogenic risk was determined by the following equation.^37^

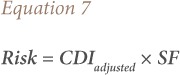
Where Risk = probability of carcinogenic effect (unitless) and SF = cancer slope factor (mg kg^−1^ day^−1^)^−1^. For non-cancer risk, the hazard quotient (HQ) was calculated using Equation 8.^37^

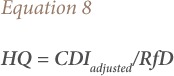
Where RfD = reference dose (mg kg^−1^ day^−1^).

## Results

Table 1 shows the descriptive statistics of the metal concentrations and the determined physicochemical parameters, as well as the Dutch^38^ and Canadian^39^ soil quality guidelines for heavy metal in soils. The pH of soil samples in the study area ranged from 6.00 to 13.95. The electrical conductivity of the soil showed minimum and maximum values of 49.8 and 883.0 μS/cm, respectively, with a mean value of 381.3 μS/cm, which indicates the soils have high concentrations of inorganic ions including metals that will also increase salinity of the soil. The organic carbon content in the soils ranged between 1–19%.

**Table 1:** Descriptive Statistics of Metal Concentrations (mg/kg) and Physicochemical Parameters of Soil Samples

	Min	Max	Mean	SD	Median	95^th^ %tile	CCME^39^	VROM^38^
As	3.5	862.5	179.4	22.7	146.4	652.1	12.0	55.0
Cr	17.8	178.3	67.8	5.3	39.2	161.1	63.0	380.0
Cu	9.7	140.1	41.3	3.1	33.6	84.8	64.0	190.0
Fe	9879	77743	34633	216	26056	69238		
Mn	312.6	4393.0	1347.0	102.8	1153.0	2910		
Ni	8.0	118.1	38.7	3.3	22.3	97.2	45.0	210.0
Pb	3.6	63.2	17.6	1.4	16.7	34.6	200.0	720.0
Zn	0.5	56.1	26.3	2.1	23.1	53.87	12.0	55.0
pH	6.00	13.95	8.02	1.3	7.76			
EC (μS/cm)	49.8	883.0	381.3	23.6	337.0			
%TOC	1.10	18.94	5.08	3.69	4.67			

Abbreviations: CCME, Canadian Council of Ministers of the Environment; SD, standard deviation; TOC, total organic carbon; VROM, Ministry of Housing, Spatial Planning and the Environment.

### Total metal concentrations

**Figure 2:**
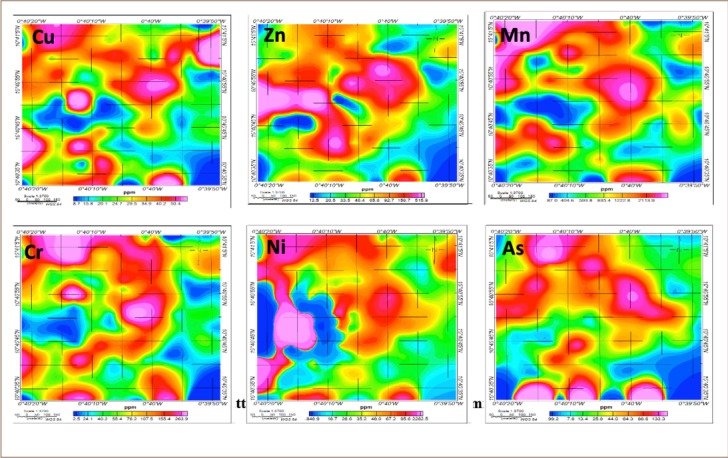
Distribution patterns of heavy metals in the Gbani community

Arsenic concentrations ranged from 3.5 to 862.5 mg/kg (Table 1), with higher concentrations recorded at the southern and northern parts of the community. Concentrations of Cr ranged from 17.8 to 178.3 mg/kg and were higher at the northern part of the community. Chromium is usually found at industrial and mining areas.^40^ The distribution of metals in the community is shown in Figure 2.

### Data comparison

Table 2 shows the regression parameters for the comparison between data from fundamental parameters-x-ray fluorescence and ICP-MS. The comparison of the two datasets shows a strong linear correlation between the two techniques for many of the metals, including Cu, Ni, As, Mn and Fe. The Pearson correlation coefficients (p<0.001) for As, Cu, Ni, Mn and Fe were 0.97, 0.77, 0.79, 0.79 and 0.70, respectively. The coefficient of determination values for As, Cu, Ni, Mn and Fe were between 0.70 and 0.97, showing excellent comparability between the XRF and ICP-MS data.^17^ The slopes of the regression lines for As, Cu, Ni, Mn and Fe were between 0.73 and 1.40, suggesting that very little data correction is needed to match the XRF data to that of the ICP-MS.^41^

**Table 2:** Regression Parameters for Fundamental Parameters-X-Ray Fluorescence and ICP-MS Comparability

Element	Coefficient of determination	Y-intercept	Slope	P-value
As	0.97	−15.79	1.40	0.00
Cr	0.41	27.97	0.30	0.00
Cu	0.77	−2.60	1.03	0.00
Fe	0.70	6916	0.73	0.00
Ni	0.79	−7.03	0.85	0.00
Mn	0.79	170	0.91	0.00
Zn	0.13	37.4	−0.17	0.22

## Discussion

pH is a major factor that influences metal persistence and its mobility in soil.^42^ Soil pH has strong effects on the solubility and retention of metals in soil; greater retention and lower solubility of a metal occur at high soil pH. Therefore, high concentrations of metals are generally expected with high pH values recorded for soil. As shown in Figure 2, metals such as Cu, Ni, Zn, and Mn which are usually associated with anthropogenic activities such as mining showed similar distribution patterns in the study area and recorded high concentrations at the northern, eastern, and central parts of the community. There are large-scale mining industry activities and automobile workshops located at the northern part of the community. Concentrations of metals recorded in this study were higher than those found in some other areas in the country such as Tarkwa and Dunkwa-on-Offin, which have similar ongoing mining activities.^43^,^44^ Concentrations of As, Cr, Cu, Mn and Ni found in the area were higher than the world average.^45^ These high concentrations of metals, especially As, could be attributed to the widespread artisanal mining activities in the community.

### Correlation between metals and physicochemical parameters

Table 3 shows the correlations between the various measured parameters. Strong positive correlations were observed between Pb, Cr, Fe, and Mn as well as Ni, Cr, and Cu, while weak positive correlations were observed between As, Cu, Cr, Ni and Pb. Correlation analyses provide an effective way to reveal the relationships between multiple variables and thus have been helpful in understanding sources of chemical components and their influencing factors. Metals in soils usually have complicated relationships between them. The high positive and significant correlations found between the metals suggest that they accumulated from similar sources. There was a strong positive correlation between pH and all of the metals, indicating the influence of pH on the availability of metals in soil. Electrical conductivity only correlated well with As, Cu, Fe, and Pb. Total organic carbon content only showed a weak positive correlation with Zn (r = 0.105) and showed a negative correlation with the other metals. This means that the organic carbon present in the soil does not have significant influence on the metal concentrations.

**Table 3:** Correlation Analysis for Metals and Physicochemical Parameters

	As	Cr	Cu	Fe	Mn	Ni	Pb	Zn	pH	EC	TOC
As	1										
Cr	0.365	1									
Cu	0.261	0.529^*^	1								
Fe	0.462^*^	0.946^**^	0.628^**^	1							
Mn	0.211	0.880^**^	0.473^*^	0.894^**^	1						
Ni	0.399	0.800^**^	0.877^**^	0.827^**^	0.736^**^	1					
Pb	0.371	0.742^**^	0.332	0.795^**^	0.877^**^	0.550^*^	1				
Zn	−0.489^*^	−0.476^*^	−0.447^*^	−0.486^*^	−0.422	−0.567^**^	−0.384	1			
pH	0.193	0.273	0.137	0.281	0.180	0.201	0.179	−0.210	1		
EC	0.453^*^	−0.028	0.012	0.023	−0.118	−0.041	0.003	−0.076	−0.076	1	
TOC	−0.135	−0.132	−0.147	−0.095	−0.025	−0.089	−0.076	0.105	−0.188	−0.114	1

^*^Correlation is significant at the 0.05 level (2-tailed)

^**^Correlation is significant at the 0.01 level (2-tailed)

Abbreviations; EC, electrical conductivity; TOC, total organic carbon

### Principal component analysis of heavy metals in the study area

Principal component analysis was performed to explore the possible sources of metals after the data was transformed using varimax rotation. Eigen values > 1 were only considered for interpreting the results (*Table 4*). Three principal components (PC) were identified which collectively accounted for 84.3% of the overall variation using varimax rotation. The PC1 constituted by Cr, Fe, Mn and Ni explained 54.7% of the overall variance, and PC2 constituted by Cu and Zn (0.45–0.80) contributed 17.8% of the total variance. Arsenic constituted PC3 (0.88) and accounted for 11.8% of the variance. The PC1 indicates anthropogenic origin for the metals, PC2 shows aerial deposition of the metals, while PC3 is attributed to natural enrichment since As is normally associated with some ores of gold.

**Table 4:** Principal Component Analysis of Heavy Metals in the Study Area

	PC1	PC2	PC3
As	0.26	0.21	**0.88**
Cr	**0.42**	−0.14	0.15
Cu	0.34	**0.45**	−0.17
NI	**0.46**	−0.17	−0.13
Fe	**0.47**	−0.11	−0.05
Mn	**0.43**	−0.24	−0.31
Zn	0.10	**0.80**	−0.24

Eigen value	3.83	1.20	0.83
% Variance	0.55	0.20	0.12
% Cumulative	0.55	0.73	0.84

Abbreviation: PC, principal component

### Assessment of heavy metal contamination

Enrichment factors of the metals are shown in Table 5. The EF values obtained for most of the metals were greater than 1, an indication that these metals were enriched and may originate from anthropogenic sources. Chromium had an EF value of 1, meaning it may emanate from a natural source. Enrichment factor values of the other metals followed the pattern As (8.45) > Zn (3.87) > Ni (1.24) = Mn (1.24) > Cu (1.05). The contamination factors for Mn, Ni, Cu, Cr and Fe were all greater than 1, indicating the area is moderately contaminated with the metals. Zinc shows considerable contamination, while the CF of As (18.59) was the highest.

**Table 5:** Mean Enrichment Factor, Mean Contamination Factor of Metals, Risk Index, Hazard Index and Carcinogenic Risk Index for Children and Adults in the Study Area

**Metals**	**EF**	**CF**	**RI**	**MI_adult male_**	**HI_adult female_**	**HI_children_**	**CRI_adult male_**	**CRI_adult female_**	**CRI_children_**
As	8.45	18.59	185.87	0.0014	0.00147	574E-03	2.692E-06	2.895E-06	1.207E-07
Cr	1.00	2.25	4.50	0.0289	0.03092	246E-02	2.833E-05	3.050E-05	3.827E-07
Cu	1.05	1.89	9.43	2.23E-05	2.33E-05	9.06E-06			
Fe	1.00	2.18	21.77	3.91E-08	4.21E-08	2.35E-08			
Mn	1.24	2.91	29.08	6.76E-08	7.28E-08	4.06E-08			
Ni	1.24	2.60	13.02	0.000249	0.00026	0.00010	2.753E-07	2.965E-07	3.716E-09
Zn	3.87	5.43	5.43	3.66E-06	3.83E-06	1.49E-06			

Abbreviations: CF, contamination factor; CRI, carcinogenic risk index; EF, enrichment factor; HI, hazard index; RI, risk index.

### Pollution load

The pollution load index for the various points in the community ranged from 0.85 to 9.16, with an average of 3.01, indicating that the soil has a high degree of metal pollution. The northwestern part of the community is highly polluted with metals along with a few spots in the center of the community as well as the southeastern sector of the community, but the major portion of the land surface area is quite unpolluted with metal contaminants. The northern and the central parts of the community are characterized by artisanal mining activities.

### Ecological health risk

The risk index analysis gave mean values ranging from 4.50 to 185.87 (*Table 5*), an indication that the metal contaminants pose a risk of adverse effects on the health of humans and the environment. The mean potential ecological risk was calculated to be 269.09, further confirming the adverse ecological risks posed by the metal contaminants. The pollution load index and potential ecological risk index all indicated metal pollution in soil in the community. Therefore, the study area could be classified as being of high ecological risk.^5^,^15^

### *In vitro* bioaccessibility and human health risk assessment

**Figure 3:**
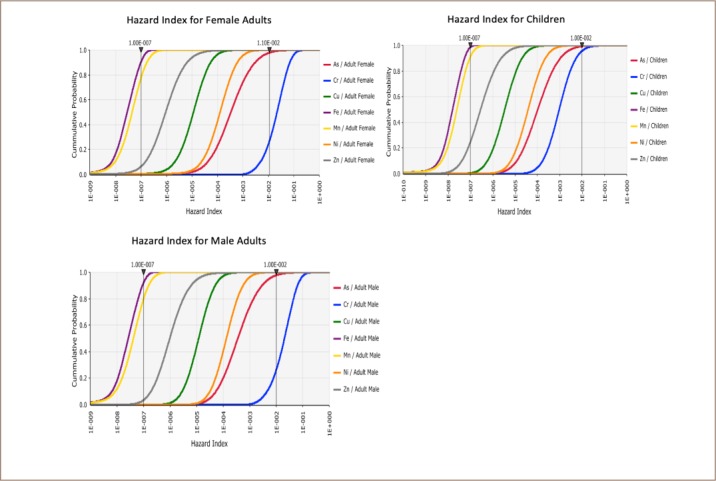
Human health risk assessments for children and female and male adults

The mean percentage bioaccessibility of the metals ranged from 3.1 to 43 (*Table 6*) based on which human health risks were modelled (*Figure 3*). Risk assessments were conducted separately for children and adults due to differences in their sensitivities to metal contamination.^16^,^26^ The use of total metal concentrations in the risk characterization indicated unacceptable risks for As and Cr, however, the adjustment of the chemical daily intake using bioaccessibility data provided a more accurate estimation of the risk associated with incidental soil ingestion. The health risks associated with incidental ingestion of the metals were generally low for adult males, adult females and children. All the risk indices were below one, indicating no adverse human health risks to adult males, adult females, or children in the community.

**Table 6:** Percentage Bioaccessibility for Total Metals

	**As**	**Cr**	**Cu**	**Fe**	**Pb**	**Mn**	**Ni**
**Mean**	8.5	5.5	36.1	3.1	41.9	43.0	22.4
**SD**	5.2	5.6	25.6	3.4	26.9	29.4	12.9
**Min**	2.7	0.3	5.6	0.5	7.4	0.0	5.3
**Max**	19.6	10.0	75.3	11.6	96.0	93.4	40.3
**Median**	6.7	3.6	29.3	1.7	39.2	35.0	21.6

Abbreviation: SD, standard deviation

## Conclusions

The present study determined levels of toxic metals (As, Cu, Ni, Cr, Zn, Fe, Mn,) in topsoil in Gbani, a mining community in Ghana. The levels of contamination showed that almost all the metals analyzed were enriched in the study area, suggesting that metals in the study area may have anthropogenic sources. Arsenic was the most enriched metal, followed by zinc. The study showed high ecological risk effects, but no associated human health risk was identified from exposure to metals at current concentrations. The soils in Gbani can be rated as moderately to highly contaminated with respect to the metals analyzed, demonstrating the need for regular monitoring of soils in the study area. Incorporating bioaccessibility results to the overall hazard indices and carcinogenic risks shows that the general risk associated with incidental ingestion of these metals in both adults and children is low.
